# Cardiac ischemia/reperfusion injury is inversely affected by thyroid hormones excess or deficiency in male Wistar rats

**DOI:** 10.1371/journal.pone.0190355

**Published:** 2018-01-05

**Authors:** Fernando A. C. Seara, Leonardo Maciel, Raiana A. Q. Barbosa, Nayana C. Rodrigues, Anderson L. B. Silveira, Michelle P. Marassi, Adriana B. Carvalho, José Hamilton M. Nascimento, Emerson L. Olivares

**Affiliations:** 1 Laboratory of Cardiovascular Physiology and Pharmacology, Department of Physiological Sciences, Institute of Biology, Federal Rural University of Rio de Janeiro, Seropedica–RJ, Brazil; 2 Laboratory of Cardiac Electrophysiology, Carlos Chagas Filho Institute of Biophysics, Federal University of Rio de Janeiro–Rio de Janeiro, Rio de Janeiro, Brazil; 3 Laboratory of Cellular and Molecular Cardiology, Carlos Chagas Filho Biophysics Institute, Federal University of Rio de Janeiro–Rio de Janeiro, Rio de Janeiro, Brazil; 4 Laboratory of Physiology and Human Performance, Department of Physical Education and Sports, Institute of Education, Federal Rural University of Rio de Janeiro, Seropedica–RJ, Brazil; Emory University, UNITED STATES

## Abstract

**Aim:**

Thyroid dysfunctions can increase the risk of myocardial ischemia and infarction. However, the repercussions on cardiac ischemia/reperfusion (IR) injury remain unclear so far. We report here the effects of hypothyroidism and thyrotoxicosis in the susceptibility to IR injury in isolated rat hearts compared to euthyroid condition and the potential role of antioxidant enzymes.

**Methods:**

Hypothyroidism and thyrotoxicosis were induced by administration of methimazole (MMZ, 300 mg/L) and thyroxine (T4, 12 mg/L), respectively in drinking water for 35 days. Isolated hearts were submitted to IR and evaluated for mechanical dysfunctions and infarct size. Superoxide dismutase types 1 and 2 (SOD1 and SOD2), glutathione peroxidase types 1 and 3 (GPX 1 and GPX3) and catalase mRNA levels were assessed by quantitative RT-PCR to investigate the potential role of antioxidant enzymes.

**Results:**

Thyrotoxicosis elicited cardiac hypertrophy and increased baseline mechanical performance, including increased left ventricle (LV) systolic pressure, LV developed pressure and derivatives of pressure (dP/dt), whereas in hypothyroid hearts exhibited decreased dP/dt. Post-ischemic recovery of LV end-diastolic pressure (LVEDP), LVDP and dP/dt was impaired in thyrotoxic rat hearts, whereas hypothyroid hearts exhibited improved LVEDP and decreased infarct size. Catalase expression was decreased by thyrotoxicosis.

**Conclusion:**

Thyrotoxicosis was correlated, at least in part, to cardiac remodeling and increased susceptibility to IR injury possibly due to down-regulation of antioxidant enzymes, whereas hypothyroid hearts were less vulnerable to IR injury.

## Introduction

Thyroid hormones (TH) are known to play crucial roles in the regulation of cardiovascular system homeostasis. By binding to nuclear receptors, TH regulates the expression of several contractile and calcium-handling proteins, ion channels and sympathetic tonus [1]. As a result, cardiac inotropic, chronotropic, lusitropic and dromotropic properties, as well as vascular resistance, are directly affected by TH levels.

It has been widely recognized from clinical data that thyroid dysfunction is correlated to increased cardiovascular morbidity and mortality [2]. Overall, tachycardia and tachyarrhythmic events, cardiac hypertrophy and heart failure have been frequently reported in conditions of TH excess, such as Graves’ disease, thyroid or pituitary adenoma, and toxic multinodular goitre [3,4]. On the other hand, bradyarrhythmias, mild hypertension, impaired systolic and diastolic functions have been associated to TH deficiency [4,5].

Importantly, clinical reports have demonstrated that hyperthyroid and hypothyroid patients are more predisposed to myocardial ischemia and acute myocardial infarction (AMI), the leading cause of death among cardiovascular diseases, when compared to euthyroid patients [6,7]. In case of TH excess, coronary artery spasm and increased prothrombotic state can elicit coronary artery occlusion and myocardial ischemia [8–10]. Myocardial ischemia can be further worsened by increased cardiac workload and oxygen demand elicited by TH excess [11]. In hypothyroid patients, dyslipidaemia and increased circulating cholesterol levels are the main contributors to the formation of atherosclerotic plaque and development of coronary artery disease [3,10]. Nonetheless, decreased blood vessel density and increased arterial wall stiffness account for the declined coronary blood flow and the higher risk of myocardial ischemia, despite the decreased cardiac workload induced by TH deficiency [11–13].

Reperfusion remains the most effective therapeutic maneuver to rescue ischemic myocardium and has been widely recommended by both American Heart Association and European Society of Cardiology [14,15]. Even so, reperfusion *per se* recruits pro-apoptotic pathways that culminate in cardiomyocyte death and further infarct expansion, a condition referred to as reperfusion injury [16]. Among several pathophysiological mechanisms, Redox imbalance has been demonstrated to play a pivotal role in the progression of ischemia/reperfusion (IR) injury by promoting oxidative damage and cell apoptosis [17].

The clinical relevance of changes on TH level in the progression of IR injury and the repercussion on AMI outcomes are unclear, whereas experimental data remain conflicting so far. Administration of 3,5,3’- triiodothyronine (T3), the main cellular active thyroid hormone, has been reported to potentiate post-ischemic recovery of myocardial mechanical properties in experimental models of IR injury [18,19]. Conversely, IR-induced myocardial damage and ventricular arrhythmias have been shown to be attenuated in hypothyroid rat hearts in comparison to euthyroid and thyrotoxic rats [20,21]. Therefore, this study aimed to assess both cardiac mechanical properties, as well as the extent of myocardial damage induced by IR in rats exposed to long-term TH deficiency and excess in comparison to euthyroid rats. The potential role of antioxidant enzyme imbalance was also investigated.

## Materials and methods

### Animals

This study followed the standards and ethical guidelines of the Ethics Committee for Research of the Federal Rural University of Rio de Janeiro and of the Federal University of Rio de Janeiro. It was approved by the Ethics Committee for Research of the Federal Rural University of Rio de Janeiro and of the Federal University of Rio de Janeiro under the number IBCCF194-07/16. Furthermore, all the standards proposed by the Guide for the Care and Use of Laboratory Animals (U.S. National Institutes of Health (NIH) Publication No. 85–23, revised 1996) were observed.

### Experimental protocol

Male Wistar rats (8 weeks-old, 170–200 g) obtained at the Central Vivarium (Carlos Chagas Filho Institute of Biophysics, UFRJ, Brazil) were housed in cages under controlled temperature (21 ± 2°C), daily exposed to 12-hour light–dark cycle (lights off at 7:00 pm) and water and standard chow *ad libitum*. The animals were divided into three groups: euthyroid (CTL, N = 18), hypothyroid (MMZ, N = 14) and thyrotoxic (T4, thyroxine group, N = 17). Hypothyroidism and thyrotoxicosis were induced by methimazole (300 mg/L) and L-thyroxine (12 mg/L), respectively, administered in drinking water for 35 days [22]. No animal exhibited adverse symptoms during all period of vehicle, T4 and MMZ exposure. After treatments, all animals were euthanized under anesthesia with isoflurane by exsanguination, and blood samples were collected for serum T4 and T3 measurements by radioimmunoassay. Hearts were excised and weighed, and the tibia length was measured for pathological analysis. The susceptibility to IR injury was evaluated in isolated hearts using a Langedorff apparatus. Cardiac samples were collected and stored at -80°C for posterior molecular analyses.

### Ex vivo IR experiments

Methods for isolated rat heart experiments were similar to those previously described [23]. Excised hearts were weighted and placed in modified Krebs-Henseleit solution (KHS) (118 mM NaCl, 4.7 mM KCl, 1.2 mM MgSO_4_, 1.2 mM KH_2_PO_4_, 25 mM NaHCO_3_, 10 mM glucose, 1.8 mM CaCl_2_, saturated with 95% O_2_ and 5% CO_2_). Aortas were hung on the cannula of a modified Langendorff apparatus and hearts were artificially perfused with modified KHS adjusted for pH 7.4 and 37°C at a constant flow (10 mL/min). A latex balloon was inserted into the left ventricular (LV) chamber through an atrial incision to assess mechanical performance. Hearts were kept immersed in perfusion solution and baseline LV end-diastolic pressure (LVEDP) was set at 10 mmHg. After 20 min of baseline period, wherein heart rate, diastolic and systolic pressures were stable, the peristaltic pump was stopped and hearts were submitted to 30 min of global ischemia and subsequent 60 min of reperfusion. LV developed pressure (LVDP), LV systolic pressure (LVSP), LV end-diastolic pressure, LV maximal derivative of pressure (max. dP/dt) and LV minimal derivative of pressure (min. dP/dt) waveforms were recorded with a pressure transducer (PT 300, Grass Technologies). The transducer was connected to an amplifier (ML 110 ADInstruments), which was connected to an analogical/digital converter (PowerLab 400, ADInstruments). All recordings were digitized and stored on a computer for later analysis using the program LabChart 5.0 (ADInstruments^®^).

### Measurement of infarct size

Ventricular sections were sliced into approximately 1.5 mm from apex to base and incubated in 1% (w/v) TTC in phosphate buffer (pH 7.4) for 5 min at 37°C. All slices were placed in a 10% (v/v) formaldehyde solution for 24 h to improve contrast between stained (viable) and unstained (necrotic) tissues. Ventricle slices were placed between two glass slides and their images were digitally acquired in a scanner. Infarct size was determined using ImageJ software (NIH ImageJ: National Institute of Health, USA, version 1.22). Values were expressed as % of total ventricular area [24].

### Radioimmunoassay for total serum T4 and T3

Blood samples were collected at the end of treatment after euthanasia (35^th^ day). Samples were centrifuged at 1931.9 x*g* for 20 min, and sera were separated and stored at -20°C. Serum T3 and T4 were determined by specific Coated-Tube Radioimmunoassay kits (MP Biomedicals, LLC, USA). All the procedures were carried out following the kit recommendation.

### Quantitative polymerase-chain reaction (qPCR)

To estimate mRNA expression levels of superoxide dismutase type 1 (SOD1), SOD2, catalase, glutathione peroxidase type 1 (GPX1) and GPX3 (Table 1), total RNA was extracted from LV tissue samples using RNeasy® Fibrous Tissue Mini Kit (QIAGEN) and cDNA was prepared from 1 μg of total RNA using High-Capacity Reverse Transcription kit (Thermo Fisher Scientific) according to the manufacturer’s instructions. mRNA levels of target genes (Table 1) were evaluated by qRT-PCR. Amplification reactions containing 1ng of cDNA were performed at 60°C during the annealing and extension cycles. The expression of chosen genes was normalized to GAPDH as an internal control. The quantification of selected mRNA was determined by 2^-(ΔΔCT)^ method in a Viia7 Software v1.2.4 and expressed as fold change of MMZ and T4 group compared to the control group.

**Table 1:** Primer sequences.

Targets	Forward	Reverse	Amplicon length
SOD1	TGTGTCCATTGAAGATCGTGTG	CTTCCAGCATTTCCAGTCTTTG	138 bp
SOD2	GGACAAACCTGAGCCCTAAG	CAAAAGACCCAAAGTCACGC	81 bp
GPX1	AATCAGTTCGGACATCAGGAG	GAAGGTAAAGAGCGGGTGAG	150 bp
GPX3	CAGCTACTGAGGTCTGACAG	ACTAGGCAGGATCTCCGAG	145 bp
Catalase	CAAGCTGGTTAATGCGAATGG	TTGAAAAGATCTCGGAGGCC	141 bp
GAPDH	CCATCAACGACCCCTTCATT	GACCAGCTTCCCATTCTCAG	110 bp

SOD1 and SOD2 = Superoxide dismutase types 1 and 2; GPX1 and GPX3 = glutathione peroxidase types 1 and 3; GAPDH = Glyceraldehyde 3-phosphate dehydrogenase.

### Statistical analysis

Data are presented as mean ± standard error of mean (S.E.M.). Normal statistical distribution of all data was determined by Shapiro-Wilks test (Statext v2.7, http://www.statext.com/index.php). One-way ANOVA followed by Bonferroni post-hoc test were used to compare MMZ and T4 groups to CTL group (Prism®, GraphPad). Statistical differences were considered significant when P < 0.05.

## Results and discussion

The present study provides evidence that the pathophysiological progression of myocardial IR injury can be distinctly affected by TH excess or deficiency. While thyrotoxic rat-hearts were more vulnerable to post-ischemic myocardial stunning than euthyroid rat hearts, post-ischemic recovery of mechanical properties was improved and infarct size was decreased in hypothyroid rat hearts. Increased susceptibility to redox imbalance and oxidative damage might contribute to these deleterious effects, given that thyrotoxic rat-hearts exhibited decreased mRNA expression level of antioxidant enzymes SOD2 and catalase.

TH deficiency is the most commonly diagnosed thyroid dysfunction and can result from several different environmental and physiological disturbances, such as iodine deficiency, primary atrophic hypothyroidism and Hashimoto’s thyroiditis [25]. The most frequent causes of TH excess are Graves’ disease, toxic multinodular goitre and thyroid adenoma [25]. Despite the limitations to mimic all features of human thyroid dysfunctions, delivery of MMZ and T4 in drinking water have been frequently used as non-invasive experimental models of hypothyroidism and thyrotoxicosis, respectively [22]. MMZ inhibits thyroidal enzyme thyroperoxidase and incorporation of iodine into thyroglobulin, resulting in drop of T3 and T4 production, as evidenced in the MMZ group (Table 2, P < 0.05 vs. CTL group). Unsurprisingly, long-term T4 exposure increased serum level of T4 (Table 2, P < 0.01 vs. CTL group). In addition, serum T3 levels were also increased in the T4 group (Table 2, P < 0.01 vs. CTL group), given that T4 can be converted into T3 by deiodinases (DIO) type 1 and 2 in target tissues. Taken together, these findings confirm that MMZ and T4 groups developed hypothyroidism and thyrotoxicosis, respectively as expected. TH-deficient rats exhibited decreased mean body weight (Table 2, P < 0.001 vs. CTL group), in agreement with the essential role of TH in the process of musculoskeletal growth by regulating pituitary and hypothalamic growth hormone synthesis [26]. Furthermore, TH deficiency has been correlated to decreased food intake and decline on body weight gain [26]. Conversely, body weight gain was not significantly affected by long-term T4 exposure (Table 2, P > 0.05 vs. CTL group). Although previous data have demonstrated that food intake can be increased by TH excess, it has been widely recognized that metabolic rate can also be increased in such a condition, which might elicit balanced body weight gain [27].

**Table 2:** Thyroid hormone levels and biometric parameters.

Parameters	CTL	MMZ	T4
**Total T4 (μg/dL)**	4.10 ± 0.25	2.06 ± 0.13*	12.19 ± 1.35**
**Total T3 (ng/dL)**	27.86 ± 5.54	8.74 ± 0.92*	347.1 ± 36.82**
**Initial BW (g)**	160.3 ± 2.431	162.0 ± 3.209	159.6 ± 3.156
**Final BW (g)**	262.0 ± 3.804	194.4 ± 4.925***	279.4 ± 10.35
**HW (g)**	1.217 ± 0.04112	0.8520 ± 0.04352**	1.582 ± 0.07506**
**HW/BW (mg/g)**	4.287 ± 0.1180	4.382 ± 0.1818	9.913 ± 0.4274***
**HW/Tibia length (g/cm)**	0.4953 ± 0.02398	0.4338 ± 0.03431	0.6833 ± 0.03318**

T4 = thyroxine; T3 = 3,5,3’ triiodothyronine; BW = body weight; HW = heart weight. Data are mean ± S.E.M. N = 14–18.

*P<0.05

**P<0.01 and

***P<0.001 vs. CTL.

Clinical and pre-clinical data have demonstrated that TH excess can induce substantial cardiac morphological changes [28,29]. Indeed, thyrotoxic rats exhibited increased HW (Table 2, P < 0.01 vs. CTL group), HW/BW (Table 2, P < 0.001 vs. CTL group) and HW/tibia length (Table 2, P < 0.01 vs. CTL group) in comparison to euthyroid rats, suggesting development of cardiac hypertrophy. At initial phases, cardiac growth in response to TH involves proportional rates of cardiomyocyte enlargement and proliferation of other cell types, such as fibroblasts and vascular cells [30–32]. At the transcriptional level, contractile and calcium-handling proteins are up-regulated, resulting in a condition frequently referred as *physiological cardiac hypertrophy* [33,34]. As demonstrated elsewhere, TH can potentiate calcium-induced calcium release from sarcoplasmic reticulum, as well as cytosolic calcium concentration during systolic period by up-regulation of ryanodine receptor expression level [33–34]. In addition, up-regulation of sarcoplasmic Ca^2+^ ATPase, as well as down-regulation of phospholambam expression levels can potentiate calcium uptake by sarcoplasmic reticulum, which has been correlated to increased relaxation rate during diastole, and further calcium release in the following excitation-contraction cycle [33–34]. Consequently, hyperthyroid patients can present increased fractional shortening and ejection fraction compared to euthyroid patients [35]. In keeping with these evidences, baseline contractility and relaxation properties were potentiated by TH excess, as evidenced by increased LVSP (Table 3, P < 0.05 vs. CTL group), LVDP (Table 3, P < 0.05 vs. CTL group) max. dP/dt (Table 3, P < 0.001 vs. CTL group) and min. dP/dt (Table 3, P < 0.05 vs. CTL group).

**Table 3:** Baseline hemodynamic parameters.

Mean baseline parameters	CTL	MMZ	T4
LVSP (mmHg)	80.34 ± 5.086	87.28 ± 3.898	98.33 ± 3.345*
LVEDP (mmHg)	11.06 ± 0.8729	10.50 ± 0.8639	9.110 ± 0.7380
LVDP (mmHg)	70.69 ± 5.100	80.31 ± 4.717	89.22 ± 3.226*
Max. dP/dt (mmHg/s)	2709 ± 130.3	1825 ± 99.36***	3517 ± 94.76***
Min. dP/dt (mmHg/s)	-1860 ± 78.35	-1462 ± 57.27*	-2205 ± 119.9*

LVSP = left ventricle systolic pressure; LVEDP = left ventricle end-diastolic pressure; LVDP = left ventricle developed pressure; max. dP/dt = maximal derivative of pressure; min. dP/dt = minimal derivative of pressure. Data are mean ± S.E.M. N = 4–6 per group.

*P < 0.05, and

***P < 0.001 vs. CTL group.

On the other hand, there is no consensus regarding cardiac structural changes elicited by TH deficiency. In general, clinical studies have demonstrated no significant changes on LV structure of hypothyroid patients [36–38]. Even so, cardiac atrophy has been reported in few experimental clinical studies, whereas cardiac hypertrophy and dilation can be observed during the progression towards heart failure [39]. Corroborating these evidences, TH-deficient rats did not show significant changes in relative HW/BW and HW/tibia length (Table 3, P > 0.05 vs. CTL group) in comparison to euthyroid rats, although absolute HW was decreased (Table 2, P < 0.01 vs. CTL group). Furthermore, contractility (Table 3, P < 0.001 vs. CTL group) and relaxation (Table 3, P < 0.05 vs. CTL group) rates were significantly decreased by hypothyroidism, in agreement with the neuralgic role played by TH in the regulation of contractile and calcium-related proteins. Indeed, decreased fractional shortening, ejection fraction, stroke volume and cardiac output have been strictly correlated to TH-deficiency-related diseases [4,5].

**Fig 1:**
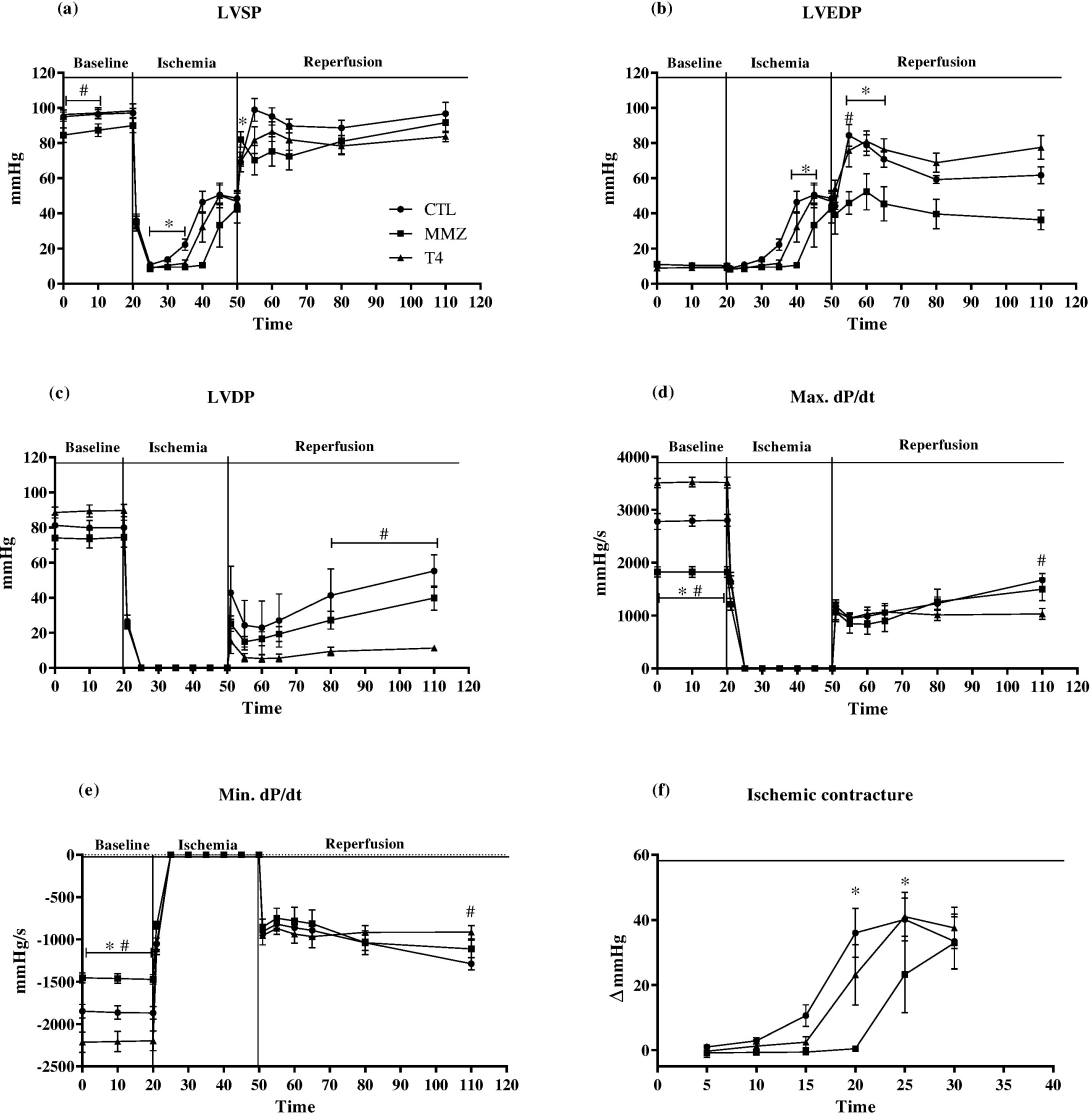
Progression of hemodynamic parameters during baseline, ischemia and reperfusion periods. Left-ventricle (LV) systolic pressure (a, LVSP), LV end-diastolic pressure (b, LVEDP), LV developed pressure (c, LVDP), maximal derivative of pressure (d, max. dP/dt), minimal derivative of pressure (e, min. dP/dt) and amplitude of ischemic contracture (f) were measured in isolated rat hearts of CTL (circles), MMZ (squares) and T4 (triangles) groups. Data are expressed as Mean ± S.E.M. *P < 0.05 vs. CTL group. N = 5–6 per group.

After the onset of ischemia, LVEDP progressively rose in all experimental groups (Fig 1). Ischemic contracture, also known as *stone heart*, has been attributed to reduced ATP bioavailability. The shift towards glycolysis and the unbalanced metabolic demand result in increased lactate production and cytosolic acidification, an effect counterbalanced by the extrusion of H^+^ by the Na^+^/H^+^ exchanger (NHX) [40]. As a result of increased NHX-induced sodium influx, the activity of Na^+^/Ca^2+^ exchanger (NCX) also increases, as does intracellular calcium concentration and LVEDP [41–43]. Interestingly, ischemic contracture was delayed (Fig 1F), whereas post-ischemic LVEDP reached the lowest levels among hypothyroid rat hearts (Table 4, P < 0.05 vs. CTL group). In agreement with our findings, Mourouzis et al. observed decreased post-ischemic LVEDP in hypothyroid rat hearts, which resulted in increased LVDP recovery [20]. In thyrotoxic rat hearts, though, LVDP recovery was significantly impaired (Table 4, P < 0.05 vs. CTL group) due to persistently increased LVEDP levels throughout the reperfusion period (Table 4, P < 0.05 vs. CTL group). Furthermore, contractility and relaxation velocities reached the lowest levels among thyrotoxic rat hearts (Table 4, P < 0.05 vs. CTL group). These findings suggest that TH excess not only worsened IR-induced myocardial contracture, but also impaired the recovery of contractility properties, a condition known as myocardial stunning.

**Table 4:** Post-ischemic hemodynamic parameters.

Reperfusion (60’)	CTL	MMZ	T4
LVSP (mmHg)	95.44 ± 6.839	91.67 ± 5.672	88.91 ± 5.588
LVEDP (mmHg)	54.21 ± 4.676	36.40 ± 5.631*	77.54 ± 6.823*
LVDP (mmHg)	41.74 ± 7.203	55.27 ± 9.185	11.37 ± 1.574*
Max. dP/dt (mmHg/s)	1700 ± 135.1	1497 ± 213.4	1032 ± 103.9*
Min. dP/dt (mmHg/s)	-1286 ± 74.02	-1111 ± 101.8	-913.8 ± 79.95*

LVSP = left ventricle systolic pressure; LVEDP = left ventricle end-diastolic pressure; LVDP = left ventricle developed pressure; max. dP/dt = maximal derivative of pressure; min. dP/dt = minimal derivative of pressure. Data are mean ± S.E.M. N = 4–6 per group.

*P < 0.05 vs. CTL group.

**Fig 2:**
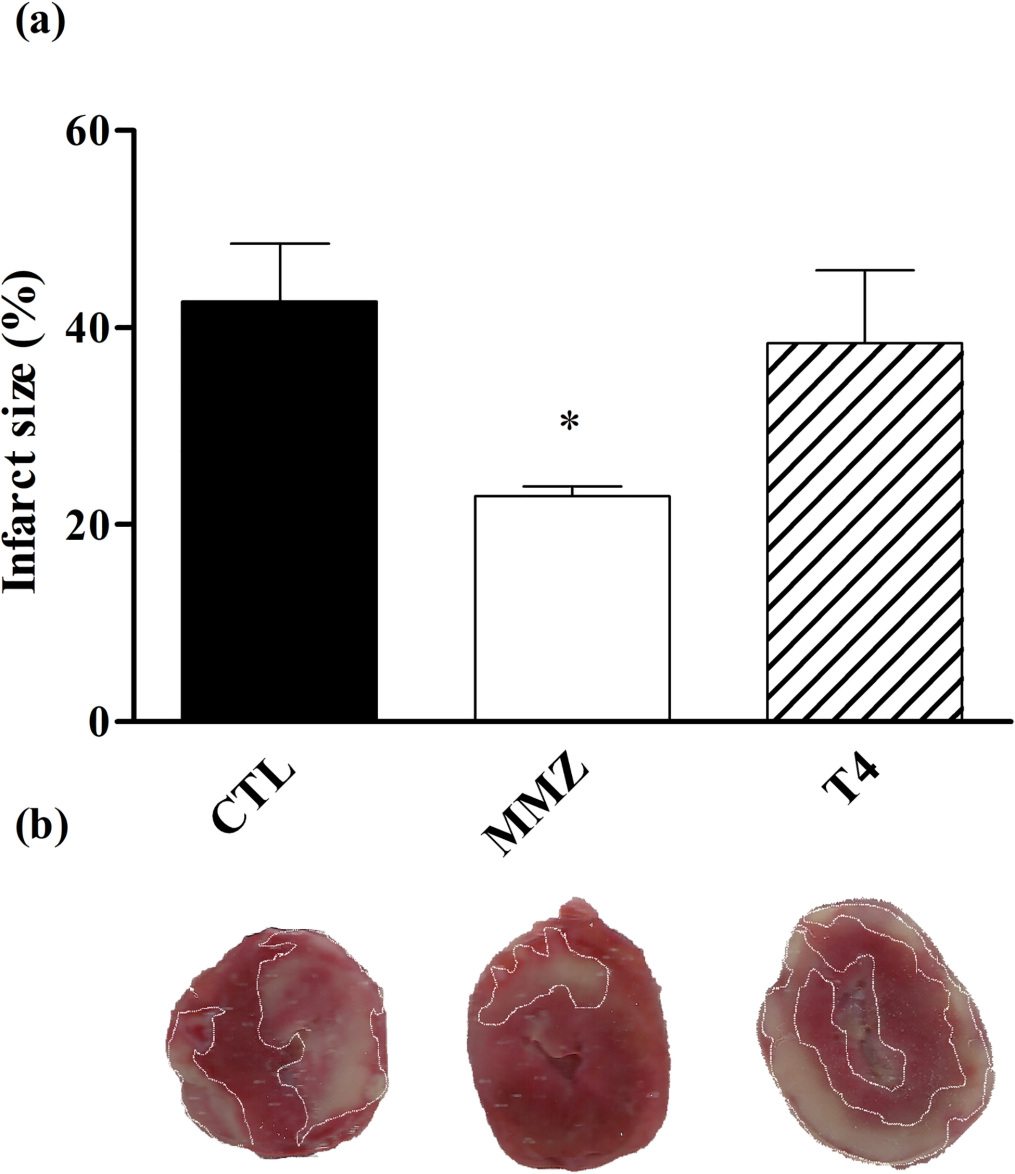
Measurement of infarct size. Percentage of infarcted area in relation to total ventricular area of CTL (black box), MMZ (white box) and T4 (striped box). Representative images of TTC-stained heart slices are shown beneath each graph box. Data are expressed as Mean ± S.E.M. *P < 0.05 vs. CTL group. N = 5–6 per group.

In contrast, acute and short-term exposure to T3 have been reported to improve IR-induced myocardial stunning in isolated rat hearts [18,19]. The increased glucose uptake induced by TH has been shown to elicit cardioprotection against doxorubicin toxicity, and this change might also elicit cardioprotection against IR injury, as evidenced by previous data [44]. At low doses or short-term exposure, TH might induce compensated cardiac hypertrophy and moderate increases on myocardial mechanical properties, consistent with TH positive inotropic and lusitropic effects [18]. However, long-term exposure to supraphysiological concentration of TH can elicit remarkable increases in ATP and oxygen consumption, which might turn thyrotoxic hearts more vulnerable to IR damage [45–47]. Conversely, hypothyroid hearts exhibited decreased metabolic rate which might be convenient in a condition marked by low bioavailability of oxygen and metabolic fuels such as ischemia [11,48]. Indeed, it has been postulated that the decreased myocardial conversion of T4 into T3 after AMI might be an adaptation to the new metabolic demand [49]. In keeping with this hypothesis, infarct size was decreased among hypothyroid rat hearts at approximately 45% compared to euthyroid rat hearts (Fig 2, P < 0.05 vs. CTL group). Furthermore, previous experimental data demonstrated reduced myocardial creatine kinase release in response to IR, which can be associated to decreased myocardial damage [20,21].

**Fig 3:**
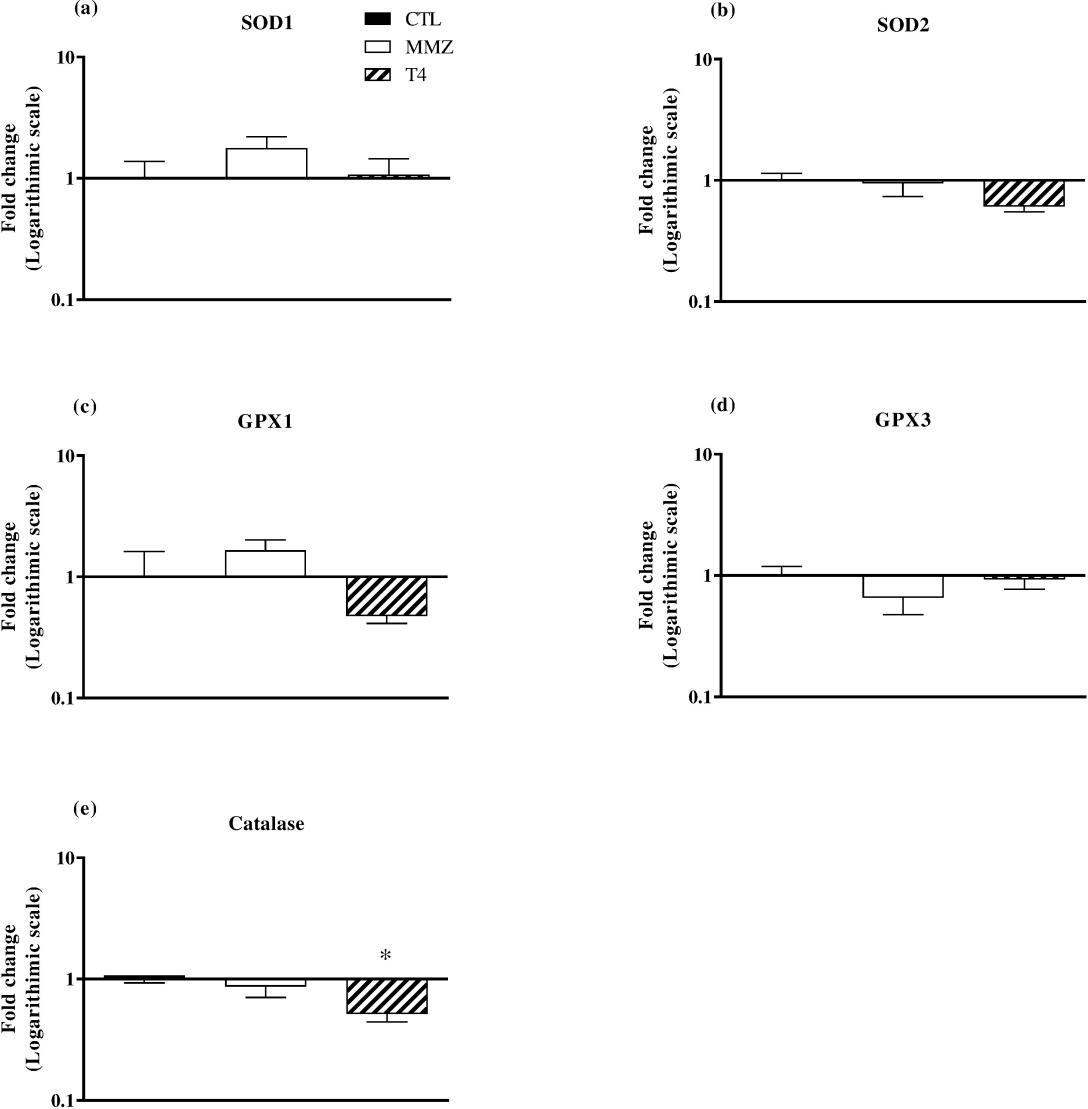
Antioxidant enzymes mRNA expression. SOD1 (a), SOD2 (b), GPX1 (c), GPX3 (d) and catalase (e) mRNA expression levels were measured in heart samples of CTL (black boxes), MMZ (white boxes) and T4 (striped boxes) groups. Data are expressed as Mean ± S.E.M. *P < 0.05 vs. CTL group. N = 5–6 per group.

The pathophysiological mechanisms involved in the opposite effects induced by TH excess and deficiency in the progression of IR injury remain unclear. However, pre-clinical studies have correlated the regulation of cellular metabolism and mitochondrial respiratory chain activity to reactive oxygen species (ROS) production and oxidative damage elicited by TH excess [45,50–54]. In a condition of redox imbalance, antioxidant enzyme provide defense against oxidative damage by promoting ROS clearance. Interestingly, catalase, SOD2 and GPX1 mRNA expression level (Fig 3) was down-regulated in thyrotoxic rat-hearts at 46.0% (P < 0.05 vs. CTL group), 37.6% (P > 0.05 vs. CTL group), and 51% (P > 0.05 vs. CTL group), respectively. On the other hand, SOD1 and GPX1 mRNA expression levels were up-regulated at 93.0% and 52.9% (Fig 3, P > 0.05 vs. CTL group), respectively, in hypothyroid rat-hearts. As previously demonstrated, SOD1, SOD2 and GPX knockout mice are more vulnerable to IR-induced damage and cell death [55–57]. On the other hand, overexpression of SOD and catalase have been correlated to post-ischemic mechanical improvements and decreased organ damage [58,59]. Previous data have also correlated decreased levels of GPX, coenzyme Q9 and Q10 to increased levels of lipid and proteins oxidative damage in thyrotoxic hearts [51–54]. Although myocardial oxygen level drops significantly after the onset of ischemia, residual oxygen level remains at approximately 3–5 Torr, which enables the production of ROS at sublethal concentration [60–62]. Nevertheless, abrupt reestablishment of myocardial oxygen level at the onset of reperfusion further increases ROS production [60]. At high concentrations, ROS can induce oxidative damage and contribute to the opening of mitochondrial permeability transition pore and release of pro-apoptotic molecules, contributing to infarct expansion [17,63]. Conversely, experimental studies have demonstrated that mitochondrial ROS production and leak at complexes I and III are reduced in hypothyroid hearts, resulting in protection against oxidative damage [45,64,65]. Therefore, redox status might play a pivotal role in the switch between increased and decreased susceptibility to myocardial IR injury elicited by TH excess and deficiency, respectively.

It remains unclear, though, how cardiac antioxidant defense was down-regulated by thyrotoxicosis. Clinical studies have reported decreased antioxidant enzymes expression levels in hyperthyroid patients, which can be restored by antithyroid drug therapy [66,67]. However, several data have demonstrated that hyperthyroidism can be followed by increased expression levels of antioxidant enzymes, as a compensatory mechanism to increased ROS production [51–54]. Noteworthy, thyrotoxic rat-hearts were also exposed to IR in the present study, suggesting that thyrotoxicosis might be followed by decreased expression levels of antioxidant enzymes particularly in conditions of physiological challenge. In addition, these findings also suggest that secondary effects, instead direct transcriptional effect of TH receptors, might play important roles in thyrotoxicosis-induced redox imbalance. Indeed, it has been demonstrated that hyperthyroidism can elicit autonomic imbalance, severe insulin resistance and unbalanced adipokine bioavailability, including decreased circulating levels of vaspin, while visfatin and resistin levels can be increased, irrespective of changes on body weight [68–70]. Together, these metabolic abnormalities can elicit cardiomyocyte pro-inflammatory changes and mitochondrial dysfunctions, which can ultimately aggravate redox imbalance and IR damage [71,72].

Taken together, the present findings demonstrated that long-term exposure to TH excess or deficiency distinctly affected the progression of myocardial IR injury. Whereas post-ischemic recovery of mechanical properties was slightly improved and infarct size decreased by TH deficiency, myocardial stunning was worsened was increased in thyrotoxic rat-hearts. These findings were correlated to decreased expression of catalase in the condition of TH excess. However, additional studies will be necessary to investigate the role of redox imbalance and cardiac remodeling in the opposite effects induced by TH deficiency or excess in the susceptibility to IR injury.

## References

[1] Kahaly GJ, Dillmann WH. Thyroid hormone action in the heart [Internet]. Endocrine Reviews. Proc 74th Annual Meeting of American Thyroid Association, Los Angeles, CA; 2005. pp. 704–728. doi: 10.1210/er.2003-0033 10.1210/er.2003-0033 15632316

[2] Parle J V, Maisonneuve P, Sheppard MC, Boyle P, Franklyn J a. Prediction of all-cause and cardiovascular mortality in elderly people from one low serum thyrotropin result: a 10-year cohort study. Lancet. 2001;358: 861–865. doi: 10.1016/S0140-6736(01)06067-6 11567699 10.1016/S0140-6736(01)06067-6

[3] Osman F, Franklyn JA, Holder RL, Sheppard MC, Gammage MD. Cardiovascular Manifestations of Hyperthyroidism Before and After Antithyroid Therapy. A Matched Case-Control Study. J Am Coll Cardiol. 2007;49: 71–81. doi: 10.1016/j.jacc.2006.08.042 17207725 10.1016/j.jacc.2006.08.042

[4] Rodondi N, Bauer DC, Cappola AR, Cornuz J, Robbins J, Fried LP, et al Subclinical Thyroid Dysfunction, Cardiac Function, and the Risk of Heart Failure. The Cardiovascular Health Study. J Am Coll Cardiol. 2008;52: 1152–1159. doi: 10.1016/j.jacc.2008.07.009 18804743 10.1016/j.jacc.2008.07.009 PMC2874755

[5] Ripoli A, Pingitore A, Favilli B, Bottoni A, Turchi S, Osman NF, et al Does subclinical hypothyroidism affect cardiac pump performance? J Am Coll Cardiol. 2005;45: 439–445. doi: 10.1016/j.jacc.2004.10.044 15680725 10.1016/j.jacc.2004.10.044

[6] Collet T-H, Gussekloo J, Bauer DC, den Elzen WPJ, Cappola AR, Balmer P, et al Subclinical Hyperthyroidism and the Risk of Coronary Heart Disease and Mortality. Arch Intern Med. American Medical Association; 2012;172: archinternmed.2012.402-. doi: 10.1001/archinternmed.2012.402 22529182 10.1001/archinternmed.2012.402 PMC3872478

[7] Ochs N, Auer R, Bauer DC, Nanchen D, Gussekloo J, Cornuz J, et al Meta-analysis: Subclinical thyroid dysfunction and the risk for coronary heart disease and mortality. Ann Intern Med. American College of Physicians; 2008;148: 832–845. doi: 10.7326/0003-4819-148-11-200806030-00225 18490668 10.7326/0003-4819-148-11-200806030-00225

[8] Patel R, Peterson G, Rohatgi A, Ghayee HK, Keeley EC, Auchus RJ, et al Hyperthyroidism-associated coronary vasospasm with myocardial infarction and subsequent euthyroid angina. [Internet]. Thyroid: official journal of the American Thyroid Association. Mary Ann Liebert, Inc. 140 Huguenot Street, 3rd Floor New Rochelle, NY 10801 USA; 2008 pp. 273–276. doi: 10.1089/thy.2007.0131 18279027 10.1089/thy.2007.0131

[9] Dörr M, Robinson DM, Wallaschofski H, Schwahn C, John U, Felix SB, et al Low serum thyrotropin is associated with high plasma fibrinogen. J Clin Endocrinol Metab. Oxford University Press; 2006;91: 530–4. doi: 10.1210/jc.2005-1786 16303831 10.1210/jc.2005-1786

[10] Erem C. Blood coagulation, fibrinolytic activity and lipid profile in subclinical thyroid disease: Subclinical hyperthyroidism increases plasma factor X activity. Clin Endocrinol (Oxf). Blackwell Publishing Ltd; 2006;64: 323–329. doi: 10.1111/j.1365-2265.2006.02464.x 16487444 10.1111/j.1365-2265.2006.02464.x

[11] Bengel FM, Nekolla SG, Ibrahim T, Weniger C, Ziegler SI, Schwaiger M. Effect of thyroid hormones on cardiac function, geometry, and oxidative metabolism assessed noninvasively by positron emission tomography and magnetic resonance imaging. J Clin Endocrinol Metab. Lippincott-Raven Press; 2000;85: 1822–7. doi: 10.1210/jcem.85.5.6520 10843159 10.1210/jcem.85.5.6520

[12] Lekakis J, Papamichael C, Alevizaki M, Piperingos G, Marafelia P, Mantzos J, et al Flow-mediated, endothelium-dependent vasodilation is impaired in subjects with hypothyroidism, borderline hypothyroidism, and high-normal serum thyrotropin (TSH) values. Thyroid. 1997;7: 411–4. doi: 10.1089/thy.1997.7.411 9226212 10.1089/thy.1997.7.411

[13] Dagre AG, Lekakis JP, Papaioannou TG, Papamichael CM, Koutras DA, Stamatelopoulos SF, et al Arterial stiffness is increased in subjects with hypothyroidism. Int J Cardiol. 2005;103: 1–6. doi: 10.1016/j.ijcard.2004.05.068 16061115 10.1016/j.ijcard.2004.05.068

[14] Wijns W, Kolh P, Danchin N, Di Mario C, Falk V, Folliguet T, et al Guidelines on myocardial revascularization: The Task Force on Myocardial Revascularization of the European Society of Cardiology (ESC) and the European Association for Cardio-Thoracic Surgery (EACTS). Eur Heart J. 2010;31: 2501–2555. doi: 10.1093/eurheartj/ehq277 20802248 10.1093/eurheartj/ehq277

[15] Levine GN, Bates ER, Bittl JA, Brindis RG, Fihn SD, Fleisher LA, et al 2016 ACC/AHA Guideline Focused Update on Duration of Dual Antiplatelet Therapy in Patients with Coronary Artery Disease: A Report of the American College of Cardiology/American Heart Association Task Force on Clinical Practice Guidelines. Circulation. 2016 doi: 10.1161/CIR.0000000000000404 27751237 10.1016/j.jtcvs.2016.07.044

[16] Braunwald E, Kloner RA. Myocardial reperfusion: A double-edged sword? J Clin Invest. American Society for Clinical Investigation; 1985;76: 1713–1719. doi: 10.1172/JCI112160 4056048 10.1172/JCI112160 PMC424191

[17] Chouchani ET, Pell VR, Gaude E, Aksentijević D, Sundier SY, Robb EL, et al Ischaemic accumulation of succinate controls reperfusion injury through mitochondrial ROS. Nature. Europe PMC Funders; 2014;515: 431–435. doi: 10.1038/nature13909 25383517 10.1038/nature13909 PMC4255242

[18] Pantos C, Malliopoulou V, Paizis I, Moraitis P, Mourouzis I, Tzeis S, et al Thyroid hormone and cardioprotection: Study of p38 MAPK and JNKs during ischaemia and at reperfusion in isolated rat heart. Mol Cell Biochem. Boston, MA: Springer US; 2003;242: 173–180. doi: 10.1023/A:1021162417490 12619880

[19] Pantos C, Mourouzis I, Saranteas T, Brozou V, Galanopoulos G, Kostopanagiotou G, et al Acute T3 treatment protects the heart against ischemia-reperfusion injury via TRα1 receptor. Mol Cell Biochem. Springer US; 2011;353: 235–241. doi: 10.1007/s11010-011-0791-8 21442236 10.1007/s11010-011-0791-8

[20] Mourouzis I, Dimopoulos A, Saranteas T, Tsinarakis N, Livadarou E, Spanou D, et al Ischemic preconditioning fails to confer additional protection against ischemia-reperfusion injury in the hypothyroid rat heart. Physiol Res. 2009;58: 29–38. doi:1387 [pii] 18198989 10.33549/physiolres.931387

[21] Bobadilla I, Franco M, Cruz D, Zamora J, Robles SG, Chávez E. Hypothyroidism provides resistance to reperfusion injury following myocardium ischemia. Int J Biochem Cell Biol. 2001;33: 499–506. doi: 10.1016/S1357-2725(01)00016-4 11331205 10.1016/s1357-2725(01)00016-4

[22] Bianco AC, Anderson G, Forrest D, Galton VA, Zs Gereben B, Kim BW, et al American Thyroid Association Guide to Investigating Thyroid Hormone Economy and Action in Rodent and Cell Models. Thyroid. Mary Ann Liebert, Inc.; 2014;24: 88–168. doi: 10.1089/thy.2013.0109 24001133 10.1089/thy.2013.0109 PMC3887458

[23] Chaves EA, Pereira-Junior PP, Fortunato RS, Masuda MO, de Carvalho ACCACC, de Carvalho DP, et al Nandrolone decanoate impairs exercise-induced cardioprotection: Role of antioxidant enzymes. J Steroid Biochem Mol Biol. 2006;99: 223–230. doi: 10.1016/j.jsbmb.2006.01.004 16621517 10.1016/j.jsbmb.2006.01.004

[24] Yamashita N, Baxter GF, Yellon DM. Exercise directly enhances myocardial tolerance to ischaemia-reperfusion injury in the rat through a protein kinase C mediated mechanism. Heart. 2001;85: 331–6. doi: 10.1136/heart.85.3.331 11179278 10.1136/heart.85.3.331 PMC1729658

[25] Vanderpump MPJ. The epidemiology of thyroid disease. Br Med Bull. Royal College of Physicians, London; 2011;99: 39–51. doi: 10.1093/bmb/ldr030 21893493 10.1093/bmb/ldr030

[26] Kamegai J, Tamura H, Ishii S, Sugihara H, Wakabayashi I. Thyroid hormones regulate pituitary growth hormone secretagogue receptor gene expression. J Neuroendocrinol. 2001;13: 275–278. doi: 10.1046/j.1365-2826.2001.00623.x 11207942 10.1046/j.1365-2826.2001.00623.x

[27] Kong WM, Martin NM, Smith KL, Gardiner J V., Connoley IP, Stephens DA, et al Triiodothyronine Stimulates Food Intake via the Hypothalamic Ventromedial Nucleus Independent of Changes in Energy Expenditure. Endocrinology. Oxford University Press; 2004;145: 5252–5258. doi: 10.1210/en.2004-0545 15297436 10.1210/en.2004-0545

[28] Bedotto JB, Gay RG, Graham SD, Morkin E, Goldman S. Cardiac hypertrophy induced by thyroid hormone is independent of loading conditions and beta adrenoceptor blockade. J Pharmacol Exp Ther. 1989;248: 632–636. 2537405

[29] Biondi B, Palmieri EA, Fazio S, Cosco C, Nocera M, Saccà L, et al Endogenous subclinical hyperthyroidism affects quality of life and cardiac morphology and function in young and middle-aged patients. J Clin Endocrinol Metab. Oxford University Press; 2000;85: 4701–4705. doi: 10.1210/jcem.85.12.7085 11134131 10.1210/jcem.85.12.7085

[30] Yao JL, Eghbali M. DECREASED COLLAGEN GENE-EXPRESSION AND ABSENCE OF FIBROSIS IN THYROID HORMONE-INDUCED MYOCARDIAL HYPERTROPHY—RESPONSE OF CARDIAC FIBROBLASTS TO THYROID-HORMONE INVITRO. Circ Res. 1992;71: 831–839. doi: 10.1161/01.RES.71.4.831 1381294 10.1161/01.res.71.4.831

[31] Tang Y Da Kuzman JA, Said S Anderson BE, Wang X Gerdes AM. Low thyroid function leads to cardiac atrophy with chamber dilatation, impaired myocardial blood flow, loss of arterioles, and severe systolic dysfunction. Circulation. 2005;112: 3122–3130. doi: 10.1161/CIRCULATIONAHA.105.572883 16275864 10.1161/CIRCULATIONAHA.105.572883

[32] Kenessey A, Ojamaa K. Thyroid hormone stimulates protein synthesis in the cardiomyocyte by activating the Akt-mTOR and p70S6K pathways. J Biol Chem. American Society for Biochemistry and Molecular Biology; 2006;281: 20666–20672. doi: 10.1074/jbc.M512671200 16717100 10.1074/jbc.M512671200

[33] Thomas TA, Kuzman JA, Anderson BE, Andersen SMK, Schlenker EH, Holder MS, et al Thyroid hormones induce unique and potentially beneficial changes in cardiac myocyte shape in hypertensive rats near heart failure. Am J Physiol Heart Circ Physiol. 2005;288: H2118–22. doi: 10.1152/ajpheart.01000.2004 15604125 10.1152/ajpheart.01000.2004

[34] Chang KC, Figueredo VM, Schreur JH, Kariya K, Weiner MW, Simpson PC, et al Thyroid hormone improves function and Ca2+ handling in pressure overload hypertrophy. Association with increased sarcoplasmic reticulum Ca2+-ATPase and alpha-myosin heavy chain in rat hearts. J Clin Invest. American Society for Clinical Investigation; 1997;100: 1742–9. doi: 10.1172/JCI119699 9312172 10.1172/JCI119699 PMC508357

[35] Ching GW, Franklyn JA, Stallard TJ, Daykin J, Sheppard MC, Gammage MD, et al Cardiac hypertrophy as a result of long-term thyroxine therapy and thyrotoxicosis. Heart. BMJ Publishing Group Ltd; 1996;75: 363–368. doi: 10.1136/hrt.75.4.363 8705762 10.1136/hrt.75.4.363 PMC484311

[36] Vitale G, Galderisi M, Lupoli GA, Celentano A, Pietropaolo I, Parenti N, et al Left Ventricular Myocardial Impairment in Subclinical Hypothyroidism Assessed by a New Ultrasound Tool: Pulsed Tissue Doppler. J Clin Endocrinol Metab. Oxford University Press; 2002;87: 4350–4355. doi: 10.1210/jc.2002-011764 12213897 10.1210/jc.2002-011764

[37] Yazici M, Gorgulu S, Sertbas Y, Erbilen E, Albayrak S, Yildiz O, et al Effects of thyroxin therapy on cardiac function in patients with subclinical hypothyroidism: index of myocardial performance in the evaluation of left ventricular function. Int J Cardiol. 2004;95: 135–143. doi: 10.1016/j.ijcard.2003.05.015 15193811 10.1016/j.ijcard.2003.05.015

[38] Pearce EN, Yang Q, Benjamin EJ, Aragam J, Vasan RS. Thyroid function and left ventricular structure and function in the Framingham Heart Study. Thyroid. Mary Ann Liebert, Inc.; 2010;20: 369–73. doi: 10.1089/thy.2009.0272 20210671 10.1089/thy.2009.0272 PMC2867586

[39] Tang Y-D, Kuzman JA, Said S, Anderson BE, Wang X, Gerdes AM. Low Thyroid Function Leads to Cardiac Atrophy With Chamber Dilatation, Impaired Myocardial Blood Flow, Loss of Arterioles, and Severe Systolic Dysfunction. Circulation. 2005;112. 10.1161/CIRCULATIONAHA.105.572883 16275864

[40] Kusumoto K, Haist J V., Karmazyn M. Na+/H+exchange inhibition reduces hypertrophy and heart failure after myocardial infarction in rats. Am J Physiol Hear Circ Physiol. 2001;280: H738—745. 10.1152/ajpheart.2001.280.2.H738 11158973

[41] Imahashi K, Schneider MD, Steenbergen C, Murphy E. Transgenic expression of Bcl-2 modulates energy metabolism, prevents cytosolic acidification during ischemia, and reduces ischemia/reperfusion injury. Circ Res. 2004;95: 734–741. doi: 10.1161/01.RES.0000143898.67182.4c 15345651 10.1161/01.RES.0000143898.67182.4c

[42] Parsons B, Szczesna D, Zhao J, Van Slooten G, Kerrick WGL, Putkey JA, et al The effect of pH on the Ca2+ affinity of the Ca2+ regulatory sites of skeletal and cardiac troponin C in skinned muscle fibres. J Muscle Res Cell Motil. 1997;18: 599–609. doi: 10.1023/A:1018623604365 9350012 10.1023/a:1018623604365

[43] Lowe JE, Jennings RB, Reimer KA. Cardiac rigor mortis in dogs. J Mol Cell Cardiol. 1979;11: 1017–1031. doi: 10.1016/0022-2828(79)90392-4 522134 10.1016/0022-2828(79)90392-4

[44] Chen YF, Kobayashi S, Chen J, Redetzke RA, Said S, Liang Q, et al Short term triiodo-l-thyronine treatment inhibits cardiac myocyte apoptosis in border area after myocardial infarction in rats. J Mol Cell Cardiol. 2008;44: 180–187. doi: 10.1016/j.yjmcc.2007.09.009 17964598 10.1016/j.yjmcc.2007.09.009 PMC2235814

[45] Asayama K, Dobashi K, Hayashibe H, Megata Y, Kato K. Lipid peroxidation and free radical scavengers in thyroid dysfunction in the rat: A possible mechanism of injury to heart and skeletal muscle in hyperthyroidism. Endocrinology. Oxford University Press; 1987;121: 2112–2118. doi: 10.1210/endo-121-6-2112 2824181 10.1210/endo-121-6-2112

[46] Seymour AML, Eldar H, Radda GK. Hyperthyroidism results in increased glycolytic capacity in the rat heart. A 31P-NMR study. BBA—Mol Cell Res. 1990;1055: 107–116. doi: 10.1016/0167-4889(90)90110-Y 10.1016/0167-4889(90)90110-y 2242380

[47] Nishiki K, Erecińska M, Wilson DF, Cooper S. Evaluation of oxidative phosphorylation in hearts from euthyroid, hypothyroid, and hyperthyroid rats. Am J Physiol. 1978;235: C212–219. doi: 10.1152/ajpcell.1978.235.5.C212 215035 10.1152/ajpcell.1978.235.5.C212

[48] Athéa Y, Garnier A, Fortin D, Bahi L, Veksler V, Ventura-Clapier R. Mitochondrial and energetic cardiac phenotype in hypothyroid rat. Relevance to heart failure. Pflugers Arch Eur J Physiol. Springer-Verlag; 2007;455: 431–442. doi: 10.1007/s00424-007-0307-2 17638011 10.1007/s00424-007-0307-2 PMC4710782

[49] Olivares EL, Marassi MP, Fortunato RS, Da Silva ACM, Costa-E-Sousa RH, Araújo IG, et al Thyroid function disturbance and type 3 iodothyronine deiodinase induction after myocardial infarction in rats—A time course study. Endocrinology. Raven Press, 2nd ed. New York; 2007;148: 4786–4792. doi: 10.1210/en.2007-0043 17628010 10.1210/en.2007-0043

[50] Hernandez-Esquivel L, Pavon N, Buelna-Chontal M, Gonzalez-Pacheco H, Belmont J, Chavez E. Cardioprotective properties of citicoline against hyperthyroidism-induced reperfusion damage in rat hearts. Biochem Cell Biol. 2015;93: 185–191. doi: 10.1139/bcb-2014-0116 25589288 10.1139/bcb-2014-0116

[51] Venditti P, Pamplona R, Portero-Otin M, De Rosa R, Di Meo S. Effect of experimental and cold exposure induced hyperthyroidism on H 2O2 production and susceptibility to oxidative stress of rat liver mitochondria. Arch Biochem Biophys. 2006;447: 11–22. doi: 10.1016/j.abb.2006.01.008 16487474 10.1016/j.abb.2006.01.008

[52] Venditti P, De Rosa R, Caldarone G, Di Meo S. Effect of prolonged exercise on oxidative damage and susceptibility to oxidants of rat tissues in severe hyperthyroidism. Arch Biochem Biophys. 2005;442: 229–237. doi: 10.1016/j.abb.2005.08.015 16197916 10.1016/j.abb.2005.08.015

[53] Venditti P, Pamplona R, Ayala V, De Rosa R, Caldarone G, Di Meo S. Differential effects of experimental and cold-induced hyperthyroidism on factors inducing rat liver oxidative damage. J Exp Biol. 2006;209: 817–25. doi: 10.1242/jeb.02045 16481571 10.1242/jeb.02045

[54] Venditti P, De Rosa R, Portero-Otin M, Pamplona R, Di Meo S. Cold-induced hyperthyroidism produces oxidative damage in rat tissues and increases susceptibility to oxidants. Int J Biochem Cell Biol. 2004;36: 1319–1331. doi: 10.1016/j.biocel.2003.11.005 15109575 10.1016/j.biocel.2003.11.005

[55] Schneider MP, Sullivan JC, Wach PF, Boesen EI, Yamamoto T, Fukai T, et al Protective role of extracellular superoxide dismutase in renal ischemia/reperfusion injury. Kidney Int. NIH Public Access; 2010;78: 374–81. doi: 10.1038/ki.2010.141 20505656 10.1038/ki.2010.141 PMC3888358

[56] Kim GW, Kondo T, Noshita N, Chan PH. Manganese Superoxide Dismutase Deficiency Exacerbates Cerebral Infarction After Focal Cerebral Ischemia/Reperfusion in Mice. Stroke. 2002;33. 10.1161/hs0302.103745 11872908

[57] Yoshida T, Maulik N, Engelman RM, Ho Y-S, Das DK. Targeted Disruption of the Mouse Sod I Gene Makes the Hearts Vulnerable to Ischemic Reperfusion Injury. Circ Res. 2000;86. 10.1161/01.res.86.3.264 10679476

[58] Li G, Chen Y, Saari JT, Kang YJ. Catalase-overexpressing transgenic mouse heart is resistant to ischemia- reperfusion injury. Am J Physiol. 1997;273: H1090–5. doi: 10.1152/ajpheart.1997.273.3.H1090 9321793 10.1152/ajpheart.1997.273.3.H1090

[59] Tanaka M, Mokhtari GK, Terry RD, Balsam LB, Lee KH, Kofidis T, et al Overexpression of human copper/zinc superoxide dismutase (SOD1) suppresses ischemia-reperfusion injury and subsequent development of graft coronary artery disease in murine cardiac grafts. Circulation. 2004;110 doi: 10.1161/01.CIR.0000138390.81640.54 15364863 10.1161/01.CIR.0000138390.81640.54

[60] Vanden Hoek TL, Li C, Shao Z, Schumacker PT, Becker LB. Significant Levels of Oxidants are Generated by Isolated Cardiomyocytes During Ischemia Prior to Reperfusion. J Mol Cell Cardiol. 1997;29: 2571–2583. doi: 10.1006/jmcc.1997.0497 9299379 10.1006/jmcc.1997.0497

[61] Xu Y, Liu B, Zweier JL, He G. Formation of hydrogen peroxide and reduction of peroxynitrite via dismutation of superoxide at reperfusion enhances myocardial blood flow and oxygen consumption in postischemic mouse heart. J Pharmacol Exp Ther. 2008;327: 402–10. doi: 10.1124/jpet.108.142372 18685120 10.1124/jpet.108.142372 PMC2615247

[62] Ide T, Tsutsui H, Hayashidani S, Kang D, Suematsu N, Nakamura K, et al Mitochondrial DNA damage and dysfunction associated with oxidative stress in failing hearts after myocardial infarction. Circ Res. 2001;88: 529–535. doi: 10.1161/01.RES.88.5.529 11249877 10.1161/01.res.88.5.529

[63] Kim J-S, Jin Y, Lemasters JJ. Reactive oxygen species, but not Ca2+ overloading, trigger pH- and mitochondrial permeability transition-dependent death of adult rat myocytes after ischemia-reperfusion. Am J Physiol Heart Circ Physiol. 2006;290: H2024–34. doi: 10.1152/ajpheart.00683.2005 16399872 10.1152/ajpheart.00683.2005

[64] López-Torres M, Romero M, Barja G. Effect of thyroid hormones on mitochondrial oxygen free radical production and DNA oxidative damage in the rat heart. Mol Cell Endocrinol. 2000;168: 127–34. doi: 10.1016/S0303-7207(00)00302-6 11064159 10.1016/s0303-7207(00)00302-6

[65] Swaroop A, Ramasarma T. Heat exposure and hypothyroid conditions decrease hydrogen peroxide generation in liver mitochondria. Biochem J. 1985;226: 403–8. 3994666 10.1042/bj2260403 PMC1144726

[66] Wilson R. Free radicals and Graves’ disease: the effect of therapy. Clin Endocrinol. Blackwell Publishing Ltd; 1989;30: 429–433. doi: 10.1111/j.1365-2265.1989.tb00442.x 10.1111/j.1365-2265.1989.tb00442.x 2598476

[67] Lassoued S, Mseddi M, Mnif F, Abid M, Guermazi F, Masmoudi H, et al A comparative study of the oxidative profile in Graves’ disease, Hashimoto’s thyroiditis, and papillary thyroid cancer. Biol Trace Elem Res. Humana Press Inc; 2010;138: 107–115. doi: 10.1007/s12011-010-8625-1 20204550 10.1007/s12011-010-8625-1

[68] Yoshida T, Monkawa T, Hayashi M, Saruta T. Regulation of expression of leptin mRNA and secretion of leptin by thyroid hormone in 3T3-L1 adipocytes. Biochem Biophys Res Commun. Academic Press; 1997;232: 822–6. doi: 10.1006/bbrc.1997.6378 9126361 10.1006/bbrc.1997.6378

[69] González CR, Caminos JE, Vázquez MJ, Garcés MF, Cepeda LA, Ángel A, et al Regulation of visceral adipose tissue-derived serine protease inhibitor by nutritional status, metformin, gender and pituitary factors in rat white adipose tissue. J Physiol. Blackwell Publishing Ltd; 2009;587: 3741–3750. doi: 10.1113/jphysiol.2009.172510 19470778 10.1113/jphysiol.2009.172510 PMC2742295

[70] Caixàs A, Tirado R, Vendrell J, Gallart L, Megía A, Simón I, et al Plasma visfatin concentrations increase in both hyper and hypothyroid subjects after normalization of thyroid function and are not related to insulin resistance, anthropometric or inflammatory parameters. Clin Endocrinol (Oxf). Blackwell Publishing Ltd; 2009;71: 733–738. doi: 10.1111/j.1365-2265.2009.03546.x 19222486 10.1111/j.1365-2265.2009.03546.x

[71] Katakam PVG, Jordan JE, Snipes JA, Tulbert CD, Miller AW, Busija DW. Myocardial preconditioning against ischemia-reperfusion injury is abolished in Zucker obese rats with insulin resistance. Am J Physiol Regul Integr Comp Physiol. American Physiological Society; 2007;292: R920–R926. doi: 10.1152/ajpregu.00520.2006 17008456 10.1152/ajpregu.00520.2006

[72] Tan Y, Ichikawa T, Li J, Si Q, Yang H, Chen X, et al Diabetic downregulation of Nrf2 activity via ERK contributes to oxidative stress-induced insulin resistance in cardiac cells in vitro and in vivo. Diabetes. American Diabetes Association; 2011;60: 625–633. doi: 10.2337/db10-1164 21270272 10.2337/db10-1164 PMC3028364

